# In vitro antimicrosporidial activity of gold nanoparticles against *Heterosporis saurida*

**DOI:** 10.1186/s12917-016-0668-x

**Published:** 2016-03-03

**Authors:** Mona Saleh, Gokhlesh Kumar, Abdel-Azeem Abdel-Baki, Saleh Al-Quraishy, Mansour El-Matbouli

**Affiliations:** Clinical Division of Fish Medicine, University of Veterinary Medicine, Veterinaerplatz 1, 1210 Vienna, Austria; Zoology Department, College of Science, King Saud University, Riyadh, Saudi Arabia; Zoology Department, Faculty of Science, Beni-Suef University, Beni-Suef, Egypt

**Keywords:** Gold nanoparticles, Microsporidia, *Heterosporis saurida*, Lizardfish, EK-1 cells

## Abstract

**Background:**

Worldwide, there is a need to expand the number of drugs available to treat parasitic infections in aquaculture. One of the new materials being tested is metal nanoparticles, which have unique chemical and physical characteristics owing to their extremely small size and high surface area to volume ratio. We examined the effectiveness of gold nanoparticles against the microsporidian parasite *Heterosporis saurida*, which causes severe economic losses in lizard fish, *Saurida undosquamis* aquaculture.

**Results:**

We synthesized gold nanoparticles by chemical reduction of tetrachloroauric acid as a metal precursor. We assessed the antimicrosporidial efficacy of the nanoparticles against *H. saurida* using an in vitro screening approach, which we had developed previously using the eel kidney cell line EK-1. The number of *H. saurida* spores produced in EK-1 cells was reduced in a proportional manner to the dosage of gold nanoparticles administered. A cell metabolic activity test (MTT) indicated that the gold nanoparticles did not appear to be toxic to the host cells.

**Conclusions:**

Gold nanoparticles can act as an effective antimicrosporidial agent and hold promise to reduce disease in lizardfish aquaculture. Metal nanoparticles should be considered as an alternate choice for development of new antimicrosporidial drugs to combat disease problems in aquaculture.

## Background

*Saurida undosquamis* (Richardson, 1848) also known as Brushtooth lizardfish is fish species of the *Synodontidae* family. *S. undosquamis* is a Lessepsian migrant species distributed across the Indo-West Pacific including the Red Sea, Persian Gulf, Eastern Africa, Japan and Australia [1]. *S. undosquamis* invaded the Levant Basin of the Mediterranean Sea, from the Indo-West Pacific through the Suez Canal [2] and is considered one of the most successful colonizers of the Eastern Mediterranean, extending as far as the Aegean Sea [3]. The Mediterranean lizardfish population now has significant commercial value [4] in the eastern Mediterranean, where it is considered one of the most common species caught in the trawl fishery [5].

**Fig. 1:**
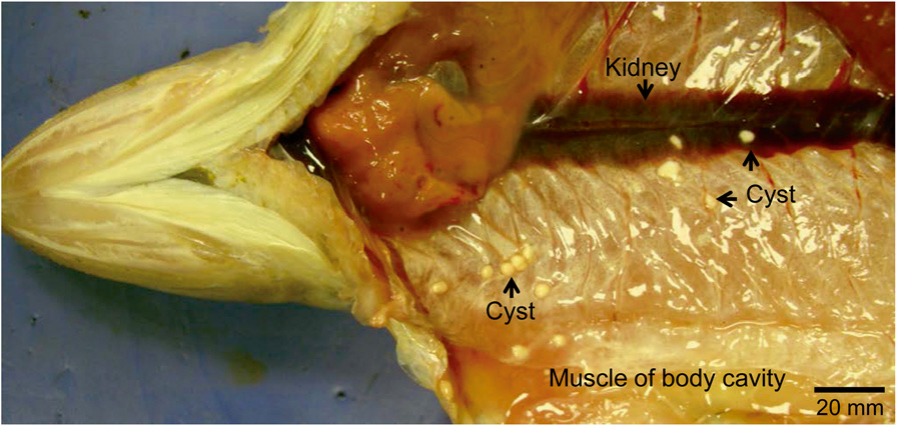
The microsporidian parasite *Heterosporis saurida* infects the lizard fish *Saurida undosquamis* and forms white, cyst-like structures in the muscles

Diseases are a major obstruction to expansion of fresh water and marine aquaculture. Fish are susceptible to many pathogens, often with severe consequences [6]. Microsporidia are single-celled, obligate intracellular parasites that infect invertebrates and vertebrates. More than one thousand microsporidian species have been recognized as causing diseases in animals and humans [7]. Microsporidia are common in marine, fresh water and estuarine systems, and affect economically important fish species worldwide, including salmonids [8], flatfish [9], greater sand eels (*Hyperoplus lanceolatus*) [10] and ornamental fish, such as zebrafish (*Danio rerio*) [11] and killifish (Family Cyprinodontidae) [12]. Infections caused by microsporidia reduce the growth rate of fish and decrease production in aquaculture [13, 14]. Recently, *H. saurida* was isolated from lizardfish (Fig. 1) where it infects skeletal muscle, body cavity and mesenteric tissues and forms white, cyst-like structures, which contain numerous spores making the fish unsuitable for public consumption [15].

Only a small number of anti-parasitic drugs are permitted in aquaculture, as the cost of drug development is high and the market small. The few available treatments for microsporidiosis differ in their effectiveness, and as with many drug treatments, there is concern for the pathogens developing resistance to one or multiple antibiotics [16]. A promising, alternative approach that has received recent attention is the use of metallic nanoparticles, which have distinct advantages over conventional antimicrobial agents [17, 18]. Nanometer-sized materials have unique, extraordinary and fascinating physical, chemical, and biological properties, particularly a large contact area with microorganisms because of their small size and higher surface-to-volume ratio; a property that expands their biological and chemical activity. Currently, nanoparticles with one dimension of 100 nm or less have received immense interest as an alternative means of combatting infectious agents in medicine. Metal based nanoparticles display broad spectrum antimicrobial activity against bacteria, fungi and viruses [19–24].

The most widespread antimicrobial compounds are silver and benzalkonium chloride [25, 26]. Unfortunately, in a comparable manner where scientists develop new efficient antimicrobial materials, there is no doubt that resistance to silver also is increasing [16]. Therefore, a number of metals, mainly copper, have been used as an antimicrobial agent. Gold has been little explored as an antimicrobial agent, although it has been used as a catalyst [27, 28].

Gold nanoparticles in particular, have a broad range of applications in nano-scale devices and technologies due to their chemical inertness and resistance to surface oxidation [29]. Gold nanoparticles have been investigated as an antimicrobial agent to inhibit the growth of common, waterborne, pathogens *Escherichia coli* and *Salmonella typhi*, which are developing resistance to common bactericides [30]. In aquaculture, several studies have been carried out to investigate gold nanoparticles as antimicrobial drugs [31–33].

In vivo efficiency trials for anti-parasitic drugs are expensive, time intensive, and need ethical permission, hence new and efficient systems to screen compounds while reducing animal experiments are required. One alternative, cost-effective approach is to screen compounds using relevant animal cell lines. In the case of *H. saurida*, in vitro propagation has been demonstrated for both mammal (rabbit kidney; [34]) and fish (eel kidney EK-1; [35]) cell lines and an in vitro screening approach using the EK-1 cells to test antimicrosporidial agents against the parasite was also developed [36], however, our aim in the present study was to investigate the in vitro antimicrosporidial activity of gold nanoparticles as a potential agent against *H. saurida* infections.

## Methods

### Ethics statement

No ethical approval was necessary as this study did not involve laboratory animals; it comprised in vitro testing of cultured cell lines and microsporidian spores.

### Propagation of *H. saurida* spores

*Heterosporis saurida* spores were grown in the eel fish kidney cell line (EK-1) using procedures previously described [35]. Briefly, *H. saurida* spores were collected from naturally infected lizard fish, *Saurida undosquamis* [15]. EK-1 cells were sub-cultured, seeded in 24-well plates in triplicate and supplemented with L-15 medium (Leibovitz) containing L-glutamine (Sigma-Aldrich), 10 % fetal bovine serum (FBS) (Sigma-Aldrich), 100 units/ml penicillin and 100 μg/ml streptomycin (Sigma-Aldrich, Vienna, Austria). Twenty four hours after seeding, 100 μl (~10^7^ spores/ml) of spore suspension was added to each well. Twenty-four hour post-inoculation (p.i.), the medium was removed and the cells rinsed gently 3 times with fresh medium; the medium was replace twice per week.

### Preparation and characterization of gold nanoparticles

We followed the protocol of Storhoff et al. [37] to synthesize gold nanoparticles (~13 nm diameter) by reduction of 10 mM tetrachloroauric acid (HAuCl_4_) using sodium citrate (Sigma-Aldrich, Vienna, Austria). Briefly, an aqueous solution of HAuCl_4_.3H2O was boiled under reflux while being stirred. The color of the solution changed from yellow to deep red after rapid addition of 10 ml 1 % trisodium citrate. The color change signified formation of monodispersed spherical gold nanoparticles. The solution refluxed for an additional 15 min, then allowed to cool to room temperature. The solution was subsequently filtered through a 0.45 μm acetate filter and stored at 4 °C. A stock concentration of 1 mg/ml gold nanoparticles was used in the assays.

Morphology, size, and shape of synthesized gold nanoparticles were characterized using transmission electron microscopy (TEM). Samples were prepared by drop casting a 2.5 mL aliquot of the Au NPs suspension onto a 300 mesh carbon-coated copper grid. The gold suspension was dried at room temperature for 5 min and overload solution was removed from the grid using blotting paper. Particles were imaged using a Zeiss EM109. The size distribution of particles was estimated by images analysis of 100 nanoparticles located at different regions of the grid (*n* = 3). Images were taken of several samples (*n* = 3) to produce statistically meaningful results.

### Measurement of anti-microsporidial activity of gold nanoparticles

Twenty-four well culture plates were loaded with EK-1 cells at a concentration of 1.5 × 10^5^ cells/ml in L-15 medium containing 10 % FBS, and penicillin-streptomycin solution (Sigma-Aldrich, Vienna, Austria). The plates were incubated overnight at 26 °C to allow a cell monolayer to form. To reach a final ratio of 3:1 spores/cell, *H. saurida* spores were added in 1 ml volumes of medium at a concentration of 10^6^ –10^7^ spores/ml. Non-adherent spores were washed off after 24 h, and fresh medium with 0, 0.01, 0.1 and 1.0 μg/ml gold nanoparticles was added to wells, and incubated for 7 days. Media was replaced every three days. Cell monolayers were examined daily with an inverted microscope, and 10 cells in 10 fields were viewed per well using a 40X objective, for each treatment group (6 wells each treatment and control). Each concentration was tested in triplicate and the inhibition of *H. saurida* spore propagation was calculated as percent inhibition = 100 – [(mean number of *H. saurida* spores counted in treated cultures/mean number of *H. saurida* spores counted in non-treated cultures) × 100]. The differences between treated and non-treated *H. saurida* spores were analyzed using *t*-tests with Bonferroni α-correction. A *p*-value < 0.05 was regarded as significant for all statistical tests. Statistical analyses were carried out using SPSS version-20 software.

### Visual observations and measurement of drug toxicity

EK-1 cells were plated in 96-well plates at approximately 1.5 × 10^4^ cells per well. After 24 h, the medium was replaced with fresh medium containing different concentrations of gold nanoparticles as described above. Cellular viability of EK-1 cultures after introduction to the gold nanoparticles was examined as above. Any toxic effects were noted, such as cells becoming sub-confluent or altered in morphology compared with non-treated control cells. Plates were incubated at 26 °C for 1 week. The medium was then removed and the viability of cultures was assayed by incubating with MTT (3-[4,5-dimethylthiazol-2-yl]-2,5-diphenyl tetrazolium bromide, Sigma-Aldrich), following the method described by Mosmann [38]: 20 μl MTT (5 mg/ml in balanced salt solution) was added to each well and incubated for 2 h. After that, medium of each well was discarded, and 200 μl of MTT solubilization solution (Sigma-Aldrich) was added and the plates agitated with an orbital shaker. Triplicate absorbance values of each well were measured in a microplate reader at 570 nm against a reference wavelength of 690 nm. The percentage of cell viability was calculated as the optical density values of treated cells divided by the mean optical density of non-treated cells, multiplied by100.

### Re-infection of EK-1 cells with *H. saurida* recovered after gold nanoparticles treatments

To determine if *H. saurida* spores were still infectious after treatment with gold nanoparticles, spores were recovered from each culture well on day 7, by addition of 100 μl of 10 % (w/v) sodium dodecyl sulfate. Released spores were centrifuged at 400 g for 15 min and washed 3 times with Tris-buffered saline containing Tween 20 (0.3 %). Spore pellets were re-suspended in medium and used to infect new cultures as previously described. A hemocytometer was used to count spores gold nanoparticles from treated and non-treated wells: 1 ml of spores suspension was adjusted to 1x10^4^ spores/ml and then added to each well. Fresh medium without gold nanoparticles was added on day 3. Each culture well was observed under an inverted microscope and spores were counted 3 times (10 field/well).

## Results

### Gold nanoparticles

**Fig. 2:**
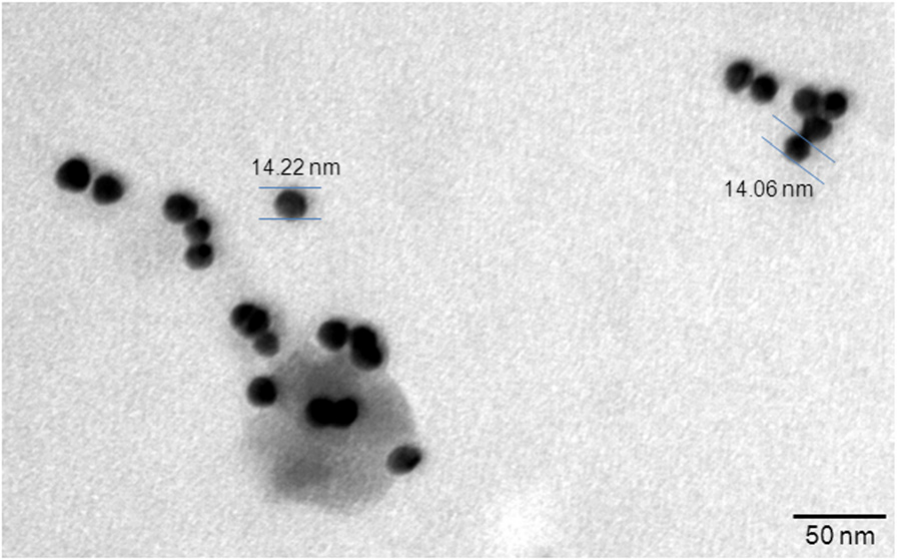
TEM micrograph of gold nanoparticles showing that most of the gold nanoparticles are round/spherical with a size range of 11.06–14.22 nm

TEM revealed that the mean diameter of gold nanoparticles was 11.06–14.22 nm, and particles were spherical (Fig. 2). TEM also showed that the gold nanoparticles suspension was in a monodispersional state without obvious aggregations.

### Measurement of anti-microsporidial activity of gold nanoparticles

**Fig. 3:**
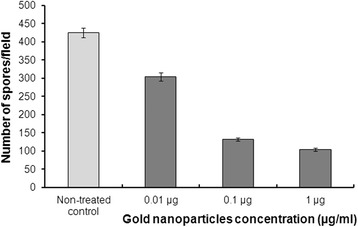
Effect of gold nanoparticles against *Heterosporis saurida* in EK-1 cells. Cells were infected with the spores of *H. saurida* and incubated with different concentrations of gold nanoparticles. Data are mean (± SD) numbers of spores in 6 wells of infected EK-1 cells

Gold nanoparticles significantly reduce (*p* < 0.001) the number of *H. saurida* spores produced by infected cells, in a concentration-dependent manner (Fig. 3). The numbers of spores observed in 10 infected EK-1 cells in 10 fields of 6 wells were recorded and are listed in Table 1. Cultures with 0.01 μg/ml gold nanoparticles had fewer *H. saurida* spores than control cell cultures. Propagation of *H. saurida* spores was inhibited 68 and 75 % by 0.1 μg/ml and 1 μg/ml gold nanoparticles, respectively. Cellular and nuclear shapes appeared normal at all gold nanoparticle concentrations.

**Table 1:** Effects of different concentrations of gold nanoparticles on proliferation of *Heterosporis saurida* and infectivity of the recovered spores in EK-1 cell cultures

Concentration of gold nanoparticles	Mean count of *H. saurida* spores	Mean percent inhibition on day 7	Cell viability (%)	Mean numbers of *H. saurida* spore (infected cells/10 fields)
0.01 μg/ml	303 ± 12.3	28.4	89.6 ± 1.7	9
0.1 μg/ml	133 ± 7.1	68.6	70.9 ± 2.2	5
1 μg/ml	104 ± 6.7	75.5	69.8 ± 1.3	3
Non-treated control cells	423 ± 15.4	-	-	67

### Drug toxicity measurements

Cell viability was monitored using MTT assay after incubation for 7 days with different concentrations of gold nanoparticles. Effects of gold nanoparticles tested concentrations were analyzed using MTT assay and results are shown in Table 1.

### Re-infection of EK-1 cells with recovered *H. saurida* spores

Since it was not appropriate to verify if these spores were mature or infectious by microscopic observation, EK-1 cells were re-infected with recovered *H. saurida* spores after treatment.

Significantly fewer infectious microsporidia spores were recovered after treatments with gold nanoparticles in a concentration-dependent manner when compared with non-treated cultures (Table 1).

## Discussion

Host- pathogen interactions are usually multivalent, and the interplay between microbes and host cells often involves several copies of multiple receptors and ligands that bind in a coordinated way to allow the microbial agent to take the cell under control. Interfering with these recognition events, by effectively crowding out pathogen entry into the cells, is one of the most promising strategies being investigated for drug development [21]. Due to the emergence of diseases and increased mortalities in aquaculture, and the development of drug resistance by aquatic microbes, there is an ongoing effort to investigate alternative methods of prevention and control of diseases [24]. A growing attention in compounds with antimicrobial characteristics is rising due to their wide application potential in various fields including medicine [39, 40].

The present study was conducted to find out if gold nanoparticles demonstrate antimicrosporidial activity against the aquatic microsporidian *Heterosporis saurida* in vitro*.*

We synthesized the gold nanoparticles (13 nm diameter) by reduction of 10 mM tetrachloroauric acid (HAuCl4) with sodium citrate.

TEM shows that most of the gold nanoparticles are round and spherical in shape and in a monodispersional state without obvious aggregations that is due to the negatively charged coating layer of citrate ions, which leads to electrostatic repulsion and prevents aggregation of gold nanoparticles.

We screened different concentrations of the nanoparticles for activity against *H. saurida* in EK-1 cells. Cell cultures treated with even the lowest concentration (0.01 μg/ml) of gold nanoparticles produced fewer *H. saurida* spores than control cell cultures (Table 1). At a concentration of 0.1 μg/ml, production of spores was inhibited more than 65 %, and at 1.0 μg/ml, inhibition was more than 75 %. The inhibitory rates obtained in this study are close to those observed when other potential antimicrosporidial drugs were used [36].

We conducted MTT assays to determine if health of host cells was adversely affected, after incubation for 7 days with different concentrations of gold nanoparticles. Negligible cytotoxicity was observed up to concentrations of 0.01 μg/ml, and < 35 % toxicity was found at the highest concentrations tested (1 μg/ml). These results are consistent with the low cytotoxicity reported previously for gold nanoparticle conjugates [41]. It has been suggested that gold nanoparticles increase permeability of the cell wall that leads to leakage of cell contents and cell death. Furthermore, gold nanoparticles have been shown to bind to the DNA of microorganisms and inhibit the DNA transcription process [41, 42]. Recently, conjugates of gold nanoparticles were investigated and reported not to be generally cytotoxic and non-specific host cell membrane disruptors but affect the transcription of a number of micro-organisms genes including those involved in cell division [43]. Similarly, in this study gold nanoparticles have been shown not to be generally toxic because the viability of the EK-1 cells was not greatly affected as confirmed with MTT test. Gene expression studies would be required to clarify the molecular mechanistic effects of gold nanoparticles on *H. saurida*, though it has been suggested that the nanoparticles may alter expression of genes related to parasite cell division [43]. The mechanism of action may also involve an increase in permeability of the cell walls, leading to leakage of cell contents and death [41, 42].

## Conclusions

This study shows that gold nanoparticles demonstrate a considerable antimicrosporidial activity against the fish microsporidian *H. saurida* in vitro. However, further investigations concerning mode of action and functionalization of gold nanoparticles to increase their antimicrosporidial activity, reduce possible in vivo accumulation related toxicity and enhance and promote their use in aquaculture are still needed.

## Abbreviations

*S. undosquamis*: *Saurida undosquamis*
*H. saurida*: *Heterosporis saurida*
EK-1: eel kidney cell line
